# Case report: Multiple arterial stenoses induced by autosomal-recessive hypophosphatemic rickets type 2 associated with mutation of ENPP1: a case study

**DOI:** 10.3389/fcvm.2023.1126445

**Published:** 2023-04-19

**Authors:** Jie Liu, Xitao Song, Daming Zhang, Yan Jiang, Mingsheng Ma, Zhengqing Qiu, Weibo Xia, Yuexin Chen

**Affiliations:** ^1^ Department of Neurosurgery, Peking Union Medical College Hospital, Chinese Academy of Medical Science and Peking Union Medical College, Beijing, China; ^2^ Department of Vascular Surgery, Peking Union Medical College Hospital, Chinese Academy of Medical Science and Peking Union Medical College, Beijing, China; ^3^ Department of Radiology, Peking Union Medical College Hospital, Chinese Academy of Medical Science and Peking Union Medical College, Beijing, China; ^4^ Department of Endocrinology, Peking Union Medical College Hospital, Chinese Academy of Medical Science and Peking Union Medical College, Beijing, China; ^5^ Department of Pediatrics, Peking Union Medical College Hospital, Chinese Academy of Medical Science and Peking Union Medical College, Beijing, China

**Keywords:** arterial stenosis, ENPP1, autosomal-recessive hypophosphatemic rickets-2, hypertension, childhood

## Abstract

Ectonucleotide pyrophosphatase/phosphodiesterase 1 (ENPP1)-related multiple arterial stenoses is a rare clinical syndrome in which global arterial calcification begins in infancy, with a high probability of early mortality, and hypophosphatemic rickets develops later in childhood. The vascular status of an ENPP1-mutated patient when they enter the rickets phase has not been thoroughly explored. In this study, we presented a case of an adolescent with an ENPP1 mutation who complained of uncontrolled hypertension. Systematic radiography showed renal, carotid, cranial, and aortic stenoses as well as random calcification foci on arterial walls. The patient was incorrectly diagnosed with Takayasu’s arteritis, and cortisol therapy had little effect on reducing the vascular stenosis. As a result, phosphate replacement, calcitriol substitution, and antihypertensive medication were prescribed, and the patient was discharged for further examination. This research presented the vascular alterations of an ENPP1-mutanted patient, and while there is less calcification, intimal thickening may be the primary cause of arterial stenosis.

## 1. Introduction

ENPP1 (ectonucleotide pyrophosphatase/phosphodiesterase 1) is a key regulator of skeletal and soft tissue mineralization (1). It is a prominent producer of extracellular inorganic pyrophosphate (PPi) and an inhibitor of fibroblast growth factor 23 (FGF23) (2); hence, biallelic loss-of-function mutations in ENPP1 are associated with two primary clinical stages: generalized arterial calcification of infancy (GACI) and autosomal-recessive hypophosphatemic rickets type 2 (ARHR2) (3). GACI caused by ENPP1 mutations may go unrecognized due to clinically insignificant vascular calcification and stenoses. ARHR2 could develop if GACI patients survived. ENPP1 also has a role in a variety of diseases such as diabetes, cancer, cardiovascular disease, and osteoarthritis (1).

ENPP1 variants were reported in 154 patients with 72.5% being demonstrably disease-causing (4), and ENPP1 deficiency prevalence was estimated approximately 1 in 64,000 pregnancies (5). It is hypothesized that both GACI and ARHR2 pathogenic theories could result in cardiovascular involvement, with distinct dominant manifestations at different ages. GACI is defined by calcification of large- and medium-sized arteries, which is coupled with intimal proliferation, and results in arterial stenosis and reduced blood flow (3, 6, 7). It has a high infant mortality rate, and affected patients may develop neonatal heart failure, arterial hypertension, and die within the first 6 months of life (8). While most children who survive to adolescence develop hypophosphatemic rickets later in childhood, and spontaneous clearance of arterial calcifications can be observed in such patients (9, 10), elevated iFGF23 levels and ARHR2 hypophosphatemia have been related to enhance survival (9). To date, it is unclear what determines whether a patient develops clinical signs of GACI or ARHR2, or both (3). Here, we present a rare instance of multiple arterial stenoses and calcification caused by an ENPP1 genetic mutation and conduct a literature review.

## 2. Case presentation

An 11-year-old Chinese boy with rickets was hospitalized to PUMCH with uncontrolled hypertension for 1 year. The boy's parents discovered his lower extremity bone abnormalities manifesting as “X-shaped legs” in 2014 (3 years old). He was also diagnosed with right renal dysplasia. Laboratory tests revealed low serum phosphorus levels and elevated ALP levels. He was, therefore, diagnosed with hypophosphatemic rickets and began treatment with oral phosphate solution and calcitriol as well as orthopedic orthosis to repair the skeletal defects. However, the treatment was ineffective. The patient, in 2021 (10 years old), requested to turn up the volume while watching TV, and a hearing test revealed bilateral hearing loss (left side worse). In July 2021 (10 years old), he had spinal kyphosis, a dragging and swinging stride, and underwent epiphyseal fixation for both knees. Prior to surgery, the brachial blood pressure was 100–110/70–80 mmHg; however, it increased to 190–200/100–110 mmHg shortly after the procedure. Palpitations, dizziness, and other discomfort symptoms were denied. Treatment with sodium nitroprusside, esmolol, and captopril could keep blood pressure at 130–140/90–100 mmHg. He was subsequently referred to have a computed tomography angiography (CTA) of renal artery, which revealed a slim right renal artery (diameter of around 1 mm) and right renal atrophy (right kidney size: 4.4 × 1.5 cm and left kidney size: 10.2 × 4.4 cm, as assessed by renal ultrasound). The left renal artery has severe stenosis in the beginning and proximal sections as well. Renal function imaging revealed that the total glomerular filtration rate (GFR) of both kidneys was 79.0 ml/min, with GFRs of 76.3 ml/min and 2.7 ml/min for the left and right kidneys, respectively. Following that, the patient was evaluated for systemic angiography, which revealed various degrees of stenosis in the right internal carotid artery (C4–7 and cervical segment) and the lower segment of the abdominal aorta (Table 1). In July 2021, the patient underwent whole exome sequencing using the next-generation sequencing method to confirm the diagnosis. The results revealed an ENPP1 mutation (c.313 + 1G > A, c.783C > G), but no mutation was detected in the ABCC6 locus. The patient was subsequently diagnosed with ARHR2. It was unclear, however, whether the multiple arterial stenoses were related to ARHR2. The patient was subsequently examined for inflammation markers at a local clinic, which revealed a minor increase ([C-reactive protein (CRP) 8.6 mg/L and erythrocyte sedimentation rate (ESR) 26 mm/h] and was suspected of having Takayasu’s arteritis. As a result, he began treatment with prednisone 75 mg q.d. and azathioprine 25 mg q.d. to slow the progression of inflammation. His antihypertensive regimen included amlodipine besylate 5 mg q.d., benazepril 5 mg b.i.d., and hydrochlorothiazide 20 mg b.i.d. Aspirin 100 mg q.d. was added for antiplatelet treatment to prevent possible thrombosis. Calcitriol 0.25 μg q.d., + calcium carbonate 0.5 g b.i.d. + vitamin D3 200 U b.i.d. were used as anti-rickets therapy. The blood pressure fluctuated between 120 and 140/70–80 mmHg while on such medication.

In December 2021, the prednisone dosage was gradually reduced to 5 mg q.d. and thereafter maintained at that level. Multiple CRP and ESR testing were normal. However, the blood pressure remained elevated at 130–140/70–80 mmHg. The patient next underwent angiography, which revealed a constricted ostial left renal artery. As a result, angioplasty was performed, and his blood pressure was decreased to 120–130/70 mmHg shortly after the procedure. While it increased to 140–150/60–70 mmHg on day 5 postoperatively. The patient was required to resume antihypertensive therapy in which the blood pressure ranged between 140 and 150/80–90 mmHg.

The patient was transferred to PUMCH in June 2022, and retested inflammatory markers were normal [ESR 6 mm/h and high sensitivity C reactive protein (hsCRP) 0.07 mg/L; complement and immunoglobulin tests, antiphospholipid antibody (APLs), antineutrophil cytoplasmic antibodies (ANCAs), and anti-nuclear antibody (ANAs) were negative]. He was measured at a height of 139 cm, which is −1 standard deviation from the average height for his age group. His weight was 38 kg, which is at 0 standard deviation from the average weight for his age group. His upper circumference was 71 cm and lower circumference was 68 cm. During examination, a systolic murmur was heard in the left renal artery but no murmur was detected in the right renal artery. There was no indication of pectus carinatum or wrist sign. His knees were valgus, and his teeth were aligned without any abnormalities in enamel development. He did not show any signs of bone tenderness throughout his body. The costo-iliac distance was measured at 4 finger widths. He was in Tanner Stage I for pubic hair development and did not exhibit any edema in both lower extremities. Right brachial artery blood pressure (156/80 mmHg) was substantially higher than other sites (left brachial artery: 133/66 mmHg, left ankle artery: 138/71 mmHg, right ankle artery: 138/64 mmHg). Laboratory tests regarding calcium and phosphorus revealed hypophosphatemia and lower but normal level of serum calcium, increased levels of alkaline phosphates, type I collagen carboxy terminal peptide β special sequence (β-CTX), hyperphosphaturia, and normal parathyroid hormone (PTH) and 25-hydroxyvitamin D (T-25OHD) (Table 3).

**Table 1:** Clinical presentations and multisystem involved in the patient.

Systems	Manifestations	Symptoms
Vascular system	Multiple vascular stenosis	Hypertension
Urinary system	Right renal atrophy	Hypertension
Bone metabolism	Hypophosphatemia	Rickets
Skeletal system	Scoliosis	Motor dysfunction
	Double genu valgum	
Auditory system	Deformity of ossicular chain	Bilateral hearing loss

**Table 2:** Stenotic arteries and calcification conditions in the patient.

Arteries	Location	Stenosis	Collateral circulation	Calcification
Left renal artery	Beginning and proximal part	Moderate-severe		Yes
Right renal artery	Whole parts	Severe(diameter = 1 mm)		
Left internal carotid artery	C1 segment	Moderate		
Right internal carotid artery	C1 segment	Occlusion	Yes	Yes
	C4–C7 segment	Mild-moderate		
Right common carotid artery	Distal part	Occlusion	Yes	
Right subclavian artery				Yes
Bilateral vertebral arteries	Medium part	Occlusion	Yes	
Bilateral middle cerebral arteries	M2 segment and distal part	Slim		
Aorta arch				Yes
Aorta. Descending	Whole parts	Slim		
Aorta. Abdominal	Whole parts	Slim	Riolan arch	
Celiac trunk	Beginning part	Severe		
Superior mesenteric artery	Beginning part	Severe		
Inferior mesenteric artery	Beginning part	Moderate		

**Table 3:** Laboratory tests regarding calcium and phosphorus.

Item	Value	Normal range	Unit
PTH	54.1	15.0–65.0	pg/ml
ALP	429	42–390	U/L
P	0.93	0.81–1.45	mmol/L
Ca	2.46	2.13–2.70	mmol/L
T-25OHD	35.6	>30	ng/ml
β-CTX	3.08	0.26–0.51	ng/ml
24hUP	29.07	—	mmol/L/24 h
24hUCa	1.01	—	mmol/L/24 h

**Figure 1:**
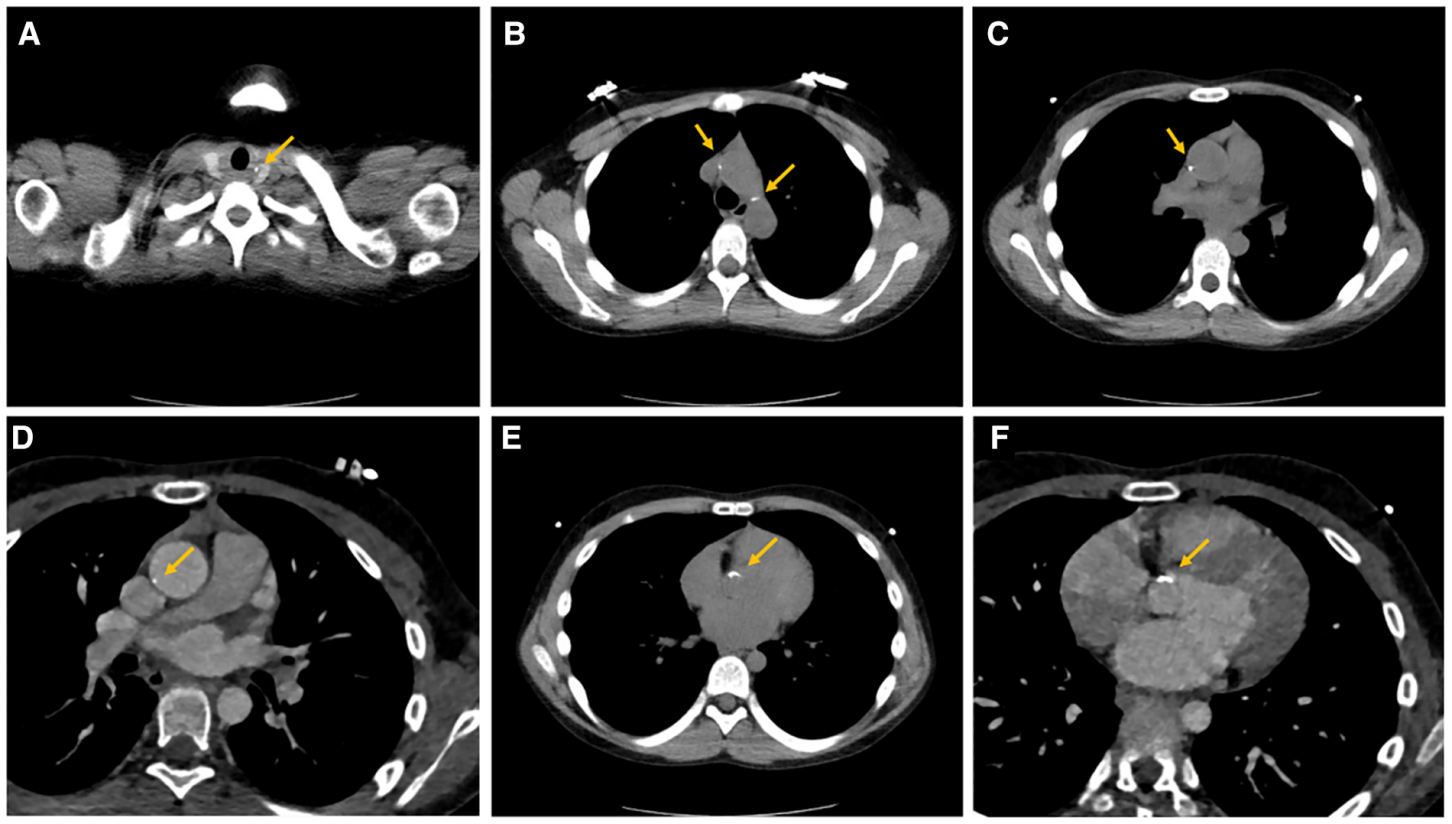
Calcifications of multiple vascular walls. Calcification foci of the aortic arch (**A,B**) and ascending aorta (**C–F**) disseminated in the arterial walls.

**Figure 2:**
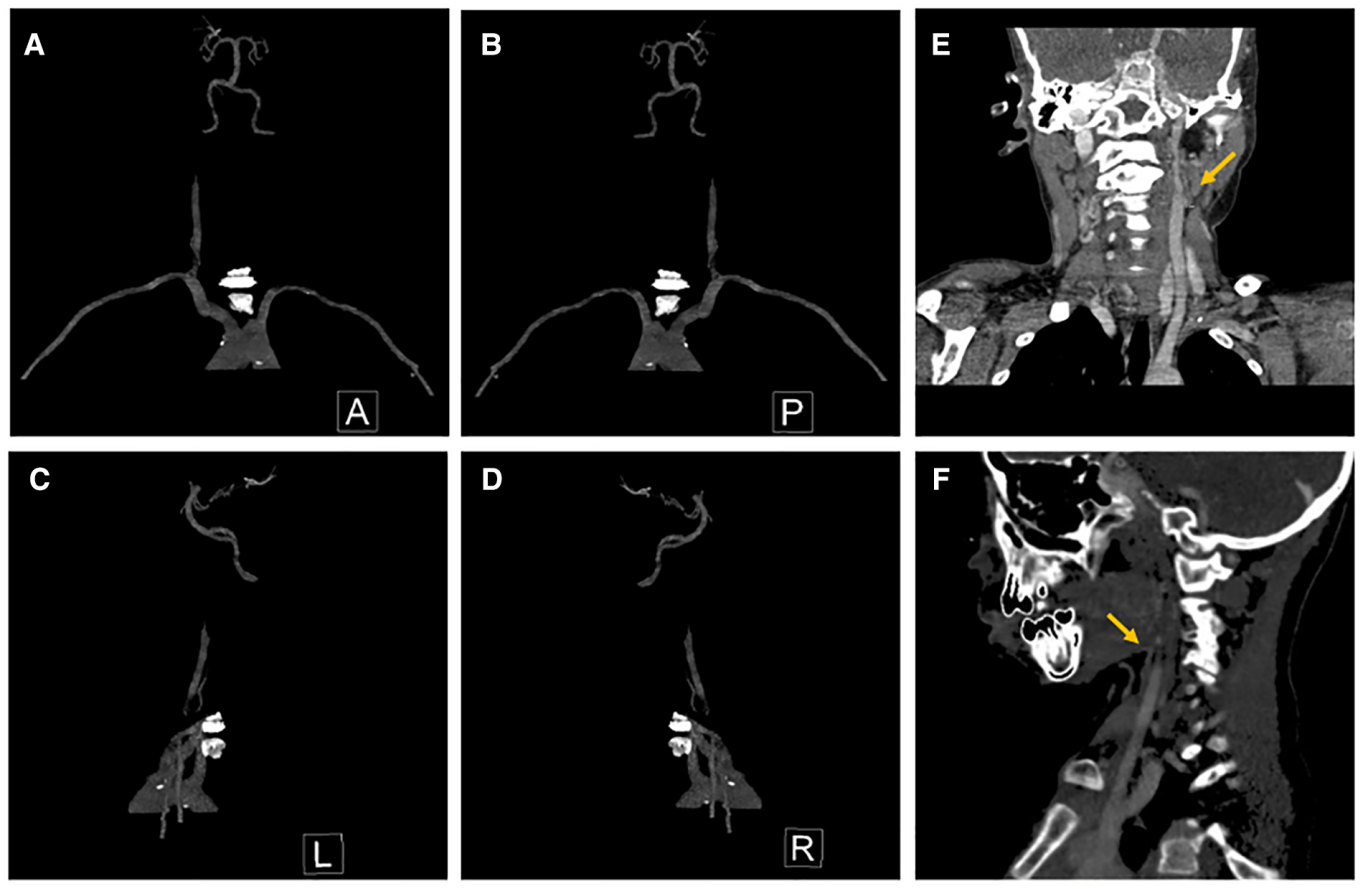
Stenosis of cranial and carotid arteries. (**A**) Anterior view, (**B**) posterior view, (**C**) lateral view (left side), (**D**) lateral view (right side), (**E**) stenosis of left ICA, and (**F**) stenosis of right ICA. ICA, internal carotid arteries.

**Figure 3:**
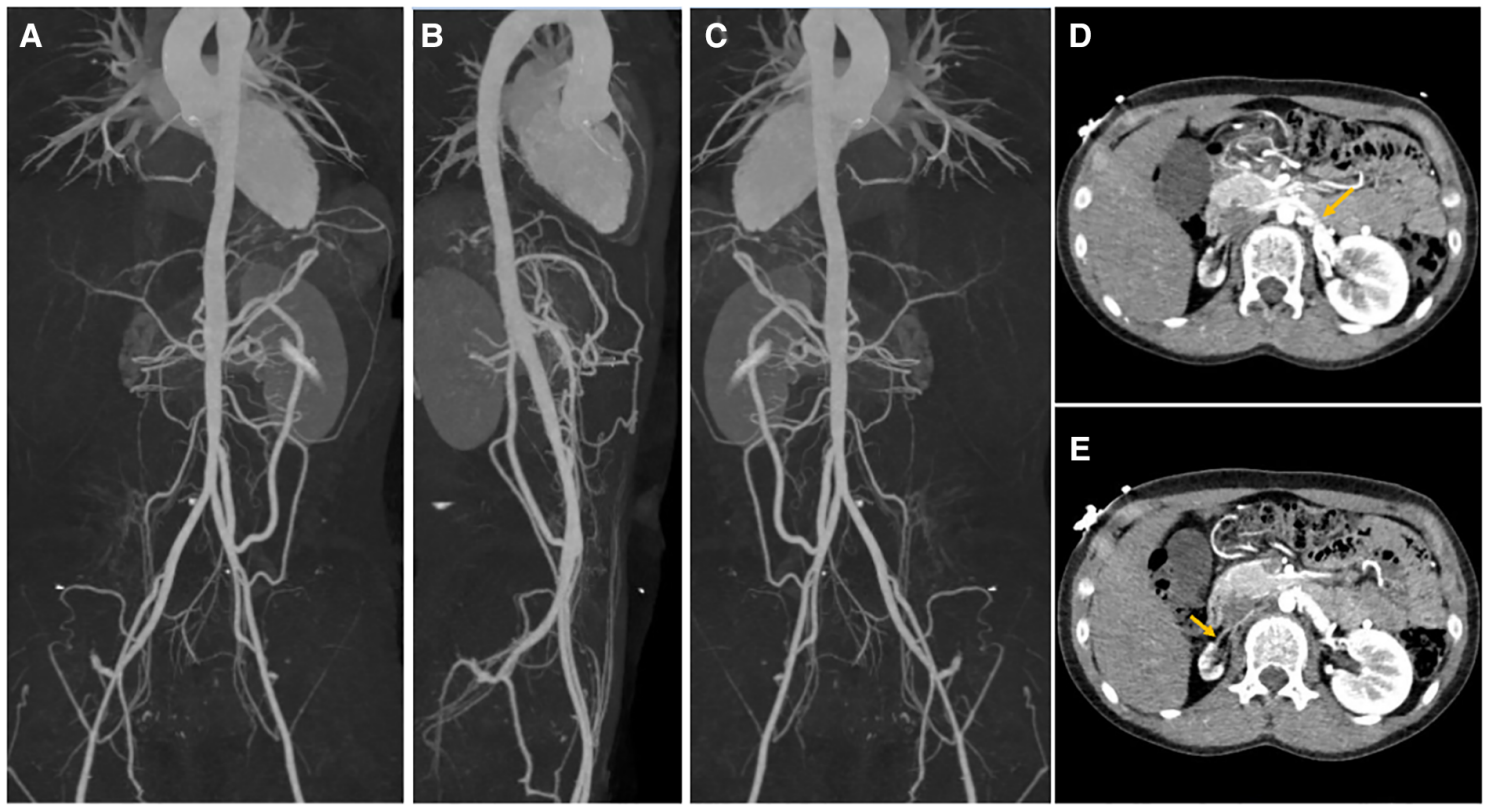
Stenosis of major and renal arteries in systematic CTA. The whole course of aorta was slender with multiple vascular thickening and stenoses. The diameter of the ascending aorta and was thoracic aorta about 21 and 12 mm, respectively. The wall of the aortic trunk and segments were thickened with calcification. Slim right renal artery was present with a diameter of about 1 mm and thickened renal arterial intima. (**A**) Anterior view, (**B**) lateral view, (**C**) posterior view of systematic CTA, (**D**) stenotic lesions of the left renal artery ostia, and (**E**) stenotic lesions of the right renal artery ostia. CTA, computed tomography angiography.

He was reexamined in July 2022 for an aorta and head CTA, which revealed calcification of the aortic arch, right subclavian artery, and right internal carotid artery (Figure 1). The descending and abdominal aortas were both thin, with numerous vascular thickening and stenosis. Severe stenosis was found in the distal segment of the right common carotid artery, the bilateral internal carotid artery (C1 segment), the proximal and middle segments of the bilateral vertebral arteries, the bilateral middle cerebral arteries (M2 segment), (Figure 2) the beginning of the left renal artery, celiac trunk, superior and inferior mesenteric arteries, and the entire course of the right renal artery. As a result, the Riolan arterial arch opened (Figure 3) (Table 2). Renal blood flow showed that the left kidney was 87.6 ml/min·1.73 m^2^ and that the right kidney did not function. Diaphragmatic ultrasound revealed that the inner diameter of the first segment of the celiac trunk did not vary significantly during the respiration process (inhale: 0.29 cm, exhale: 0.27 cm). Temporal bone CT revealed bilateral jugular bulbs reaching the cochlea, indicating ossicular chain abnormality.

The theory that arterial mineralization and calcification and proliferative alterations of arterial wall were caused by hydroxyphosphate deposition, pyrophosphate, and phosphate imbalance induced by ARHR2 could explain the arterial calcification and stenosis of various vessels. We also noticed that the inflammatory markers were not considerably elevated throughout the patient's medical history, and prednisone treatment was ineffective. Following the discussion with the multidisciplinary team (ΜDT), it was presumed that ARHR2 might be able to formulate an explanation for the full scope of the condition. The patient then reduced the cortisol dose until it was no longer needed. The patient was discharged and closely monitored after the antihypertension and phosphorous supplement strategy was adjusted.

## 3. Discussion

ENPP1-related disease is rarely documented globally, and it has a particular manifestation modality in which GACI develops in infants with a high rate of early mortality due to uncontrolled heart failure and arterial events, followed by hypophosphatemic rickets caused by ARHR2 (3). However, the disease's cardiovascular profile varies greatly. The underlying mechanism could be attributed to a transition from calcification to intimal thickening or intimal thickening may be the primary cause of multiple arterial stenoses instead of calcification, and the specific regulation of this process is still unclear. In this case, only sporadic minor calcification foci were found. After reviewing the patient’s medical records and discussion with the multidisciplinary team, we believe that at least in the adolescent period, intimal thickening, rather than calcification, was the primary cause of multiple arterial stenoses in this case.

There are few case studies and survival analyses that examine the cardiovascular changes and phosphatemic metabolisms (7–12); however, the intimal thickening changes were less noted when the patient entered the ARHR2 stage of the disease's natural history. Dlamini et al. reported three siblings with different heterozygous ENPP1 mutations from their parents (7). At 14 months, one boy had hypertension, and an arch aortogram revealed severe stenosis of the celiac axis, superior mesenteric artery, renal arteries, and both internal (ICA) and external carotid arteries, while brain CT revealed just a small region of cerebral arterial calcification. The other two siblings died perinatally due to severe cardiac and aortic calcification. This case would show that calcification is not universal and fibrointimal proliferation with subsequent vascular stenosis may occur in locations lacking calcification. Thomas et al. documented a case with prolonged survival until 11 years old and the patient also displayed discordance between the amount of vascular calcification and various arterial luminal occlusions. No phosphorus treatment was undertaken, and no genetic testing was performed (9). Ciana et al. also described two ENPP1-mutanted siblings who had numerous arterial stenoses and spontaneous clearance of arterial calcifications as they aged (10). Ferreira et al. reported an ENPP1-related newborn GACI with coronary artery blockage and calcification of the descending aorta, renal, splenic, superior mesenteric, brachial, and coronary arteries (12). Phosphate was repleted for more than 7 years and there was no progression of vascular calcification in this case (12); a retrospective observational study of 55 patients with GACI found that bisphosphonates demonstrated a trend toward benefit of improving survival (8). These cases may suggest that ENPP1-related vascular stenosis is associated with intimal thickening rather than calcification, but perinatal vascular calcification is fatal. In the children who survive to adolescence will develop hypophosphatemic rickets later and spontaneous clearance of arterial calcifications can be observed in some patients.

ENPP1 is an enzyme that converts adenosine triphosphate (ATP) to adenosine monophosphate (AMP) and PPi (13). It also suppresses FGF23 production, which is responsible for decreasing phosphate reabsorption by downregulating the expression of sodium-phosphate cotransporter in the renal proximal tubule and decreasing 1-hydroxylase activity and 1,25-dihydroxyvitamin D synthesis (14). Individuals with ENPP1 deficiency have lower amounts of PPi in their blood, which predisposes calcium and phosphorous precipitation to form calcium phosphate, notably in the vascular internal elastic lamina, cartilage, and other soft tissues (1). According to Nitschke et al., neointimal proliferation is caused by a decrease in ATP clearance and adenosine synthesis caused by ENPP1 deficiency (15), while increased FGF23 levels may be an organism's response to reduce ectopic calcification (3, 8).

In clinical practice, patients diagnosed with GACI demonstrate markedly reduced levels of systemic PPi in both their urine and plasma, with values frequently approaching zero, in contrast to the reference range documented in published studies (16). Thereafter, Bernhard et al. introduced the ATP sulfurylase method as a diagnostic tool to measure PPi levels in plasma as a potential biomarker for pediatric patients. The study examined plasma samples from 200 children between 1 day and 18 years old, who had undergone blood testing for unrelated medical conditions. The researchers established a standard range of PPi in the blood plasma of children and adolescents, aged 0–18 years, ranging from 2.36 to 4.44 µM, with a median of 3.17 µM, which did not differ by age or sex in the pediatric cohort (17).

Initially, the treatment for this condition was contentious. Since the 1960s, phosphate and calcitriol substitution have been used in conventional hypophosphatemic rickets (HR) therapy (18). Bisphosphonates, which are synthetic PPi counterparts, are used to treat bone disorders caused by excessive bone resorption and phosphate loss (19). Besides phosphate supplement to the organism, treatment with calcitriol in most forms of FGF23-dependent HR is important to balance the suppression of 1*α*-hydroxylase by FGF23, which usually results in low serum levels of 1,25-OH-D3. Clinicians have been wary of phosphate substitution because of the protective effect of FGF23 and the avoidance of additional calcification, and the traditional view sees it as a protective effect with increased survival through low phosphate levels in ENPP1 deficiency. However, phosphate treatment has been tested in animal experiments and for certain patients, with longer survival rates. In the Enpp1asj mouse model of GACI and ARHR2, the administration of bisphosphonates reduced mineralization of the epidermis and the aorta and restored the bone microarchitecture (20) Ferreira et al. reported a case of a patient with GACI who was treated for more than 7 years with phosphate (and calcitriol) to compensate for phosphate wasting with no deterioration of vascular calcification (12). Recent ARHR2 studies show that phosphate and even calcitriol substitution do not induce calcification in patients with ENPP1 impairment (11, 12). These studies revealed that phosphate treatment may be beneficial for the treatment of ENPP1 decidecncy with prolonged survival without impairing vascular calcification control.

ENPP1 enzyme replacement therapy which used recombinant Enpp1-Fc protein has been developing as a novel treatment for ENPP1-related HR and vascular lesion. ENPP1 coupled to the Fc fragment of human IgG1 has been shown in animal tests to reduce mortality and vascular calcification (21), as well as improve blood pressure and cardiac function (22). Ferreira et al. discovered that recombinant Enpp1-Fc protein replacement was beneficial for correcting low bone mass in ARHR2 mice without raising the risk of nephrocalcinosis, albeit its vascular effect has not been well investigated (23). In human research, there is currently an ongoing phase 1/2, open-label, multiple ascending dose study testing enzyme replacement therapy in adults with ENPP1 deficiency (NCT04686175) to investigate and evaluate the safety, tolerability, pharmacokinetics, and pharmacodynamics.

Surgical intervention has been rarely documented in GACI patients. The most common reason for surgery was a rapidly progressing cardiovascular event, and just a few cases were documented. Samyn et al. reported a 2-year-old child with live-born GACI-related congenital heart disease who underwent operative right ventricular outflow tract (RVOT) reconstruction and restored excellent biventricular systolic function (24). Giovannoni et al. described a case of a 4-year-old Italian kid who underwent a heart transplant at the age of 18 months due to end-stage heart failure caused by severe myocardial infarction of the left ventricle and diffuse coronary calcifications (25). To date, no surgical or interventional procedure in renal artery stenosis associated in GACI- or ENPP1-related disorders has been reported. For patients with renal artery stenosis, we have three therapeutic options: medical therapy, percutaneous therapy [including percutaneous angioplasty (PTA) and stenting], and surgical therapy (including aortorenal bypass surgery, splanchnorenal bypass surgery, and endarterectomy) (26). A clinician can select between open surgery and PTA when the treatment goal is to open a stenotic lesion (27). Percutaneous revascularization is recommended for patients of any age who have resistant or malignant HTN (26, 27). Surgical treatment may be suggested in some patients having aortic revascularization if renal revascularization cannot be performed by percutaneous procedures or has failed (27). Back to our case, the patient displayed slender right renal artery (diameter was around 1 mm) with right renal atrophy and had a history of PTA, while it did not exert the antihypertensive impact for a long time. As a result, we did not do another PTA on the patient and instead advised medical treatment.

GACI had a 54.7% overall mortality rate, with a 50.4% likelihood of death before the age of 6 months, and mortality was greater for ENPP1 variations vs. ABCC6 variants (40.5% vs. 10.5%, respectively; *p* = 0.0157) (28). In ENPP1-deficient individuals, the main morbidity in adults was related to enthesis calcification (11). Previous research in children with GACI discovered an association between hypophosphatemia and hyperphosphaturia and increased survival (8). Ferreira et al. performed a prospective long-term follow-up of 20 GACI patients (16 with homozygous ENPP1 mutation) and discovered higher iFGF23 levels in the majority of surviving GACI patients, sometimes even in the absence of clinical indications of rickets (11). These research studies demonstrated that hypophosphatemia may be a protective factor for ENPP1-deficient patients. Despite the fact that studies have demonstrated that phosphate substitution is not required to promote vascular calcification, certain patients have been reported to have a long-term survival (12, 20).

In conclusion, we present a rare case of multiple arterial stenoses in which renal artery stenosis was the primary clinical manifestation, with rickets phenotype of ARHR2 caused by genetic alterations in the ENPP1 gene and a long survival. Arteritis was effectively ruled out, and medical therapy was adjusted accordingly. We first reviewed the artery stenosis involved and verified that the stenosis of these arteries is not necessarily caused by calcification but more connected with intimal thickening. Comprehensive evaluation is required to avoid the misdiagnosis of Takayasu’s arteritis, especially when CRP and ESR disclosed modest elevation. Although it might be tried, percutaneous revascularization for renal artery stenosis was unable to lower the blood pressure in our case. Open surgery should be considered if necessary while there is no case recorded up to date. Conventional therapy including phosphate and calcitriol substitution should be taken in the ENPP1-related vascular disease and the use of ENPP1 enzyme replacement therapy (recombinant Enpp1-Fc protein) remains to be trialed in children in the future.

## Data Availability

The original contributions presented in the study are included in the article/Supplementary Material, further inquiries can be directed to the corresponding author.
